# Nesprin-1 LINC complexes recruit microtubule cytoskeleton proteins and drive pathology in *Lmna*-mutant striated muscle

**DOI:** 10.1093/hmg/ddac179

**Published:** 2022-08-04

**Authors:** Ei Leen Leong, Nyein Thet Khaing, Bruno Cadot, Wei Liang Hong, Serguei Kozlov, Hendrikje Werner, Esther Sook Miin Wong, Colin L Stewart, Brian Burke, Yin Loon Lee

**Affiliations:** Institute of Medical Biology, Agency for Science Technology and Research (A^*^STAR), 8A Biomedical Grove, Level 6 Immunos, Singapore 138648, Singapore; Department of Biological Sciences, National University of Singapore, 16 Science Drive 4, Singapore 117558, Singapore; Institute of Medical Biology, Agency for Science Technology and Research (A^*^STAR), 8A Biomedical Grove, Level 6 Immunos, Singapore 138648, Singapore; A^*^STAR Skin Research Labs (A^*^SRL), Agency for Science Technology and Research (A^*^STAR), 8A Biomedical Grove, Level 6 Immunos, Singapore 138648, Singapore; Sorbonne Université, INSERM U974, Institut de Myologie, GH Pitié Salpêtrière, 47 Boulevard de l’Hôpital, Paris 75013, France; Institute of Medical Biology, Agency for Science Technology and Research (A^*^STAR), 8A Biomedical Grove, Level 6 Immunos, Singapore 138648, Singapore; A^*^STAR Skin Research Labs (A^*^SRL), Agency for Science Technology and Research (A^*^STAR), 8A Biomedical Grove, Level 6 Immunos, Singapore 138648, Singapore; Center for Advanced Preclinical Research, Frederick National Laboratory for Cancer Research, Frederick, MD 21702, USA; Institute of Medical Biology, Agency for Science Technology and Research (A^*^STAR), 8A Biomedical Grove, Level 6 Immunos, Singapore 138648, Singapore; A^*^STAR Skin Research Labs (A^*^SRL), Agency for Science Technology and Research (A^*^STAR), 8A Biomedical Grove, Level 6 Immunos, Singapore 138648, Singapore; Institute of Medical Biology, Agency for Science Technology and Research (A^*^STAR), 8A Biomedical Grove, Level 6 Immunos, Singapore 138648, Singapore; Institute of Molecular and Cell Biology, Agency for Science, Technology and Research (A^*^STAR), 8A Biomedical Grove, Level 5 Immunos, Singapore 138648, Singapore; Institute of Medical Biology, Agency for Science Technology and Research (A^*^STAR), 8A Biomedical Grove, Level 6 Immunos, Singapore 138648, Singapore; A^*^STAR Skin Research Labs (A^*^SRL), Agency for Science Technology and Research (A^*^STAR), 8A Biomedical Grove, Level 6 Immunos, Singapore 138648, Singapore; Department of Physiology, Yong Loo Lin School of Medicine, National University of Singapore, 2 Medical Drive MD9, Singapore 117593, Singapore; Institute of Medical Biology, Agency for Science Technology and Research (A^*^STAR), 8A Biomedical Grove, Level 6 Immunos, Singapore 138648, Singapore; A^*^STAR Skin Research Labs (A^*^SRL), Agency for Science Technology and Research (A^*^STAR), 8A Biomedical Grove, Level 6 Immunos, Singapore 138648, Singapore; Institute of Medical Biology, Agency for Science Technology and Research (A^*^STAR), 8A Biomedical Grove, Level 6 Immunos, Singapore 138648, Singapore; A^*^STAR Skin Research Labs (A^*^SRL), Agency for Science Technology and Research (A^*^STAR), 8A Biomedical Grove, Level 6 Immunos, Singapore 138648, Singapore; Nuevocor Pte Ltd, 3 Biopolis Drive, #06-11 Synapse, Singapore 138623, Singapore

## Abstract

Mutations in *LMN*A*,* the gene encoding A-type lamins, cause laminopathies—diseases of striated muscle and other tissues. The aetiology of laminopathies has been attributed to perturbation of chromatin organization or structural weakening of the nuclear envelope (NE) such that the nucleus becomes more prone to mechanical damage. The latter model requires a conduit for force transmission to the nucleus. NE-associated Linker of Nucleoskeleton and Cytoskeleton (LINC) complexes are one such pathway. Using clustered regularly interspaced short palindromic repeats to disrupt the Nesprin-1 KASH (Klarsicht, ANC-1, Syne Homology) domain, we identified this LINC complex protein as the predominant NE anchor for microtubule cytoskeleton components, including nucleation activities and motor complexes, in mouse cardiomyocytes. Loss of Nesprin-1 LINC complexes resulted in loss of microtubule cytoskeleton proteins at the nucleus and changes in nuclear morphology and positioning in striated muscle cells, but with no overt physiological defects. Disrupting the KASH domain of Nesprin-1 suppresses *Lmna-*linked cardiac pathology, likely by reducing microtubule cytoskeleton activities at the nucleus. Nesprin-1 LINC complexes thus represent a potential therapeutic target for striated muscle laminopathies.

## Introduction

The type V intermediate filament proteins, lamins A, C, B1 and B2, form the nuclear lamina, a 10–20 nm thick protein meshwork lining the nuclear face of the nuclear envelope (NE) (1). Featuring inner and outer nuclear membranes (INM and ONM) separated by a ~50 nm perinuclear space (PNS) that is contiguous with the endoplasmic reticulum lumen, the NE compartmentalizes the nucleus and cytoplasm, with the nuclear lamina crucial for maintaining NE integrity (2). Mutations in NE genes, in particular *LMNA,* encoding lamins A and C, result in multiple disorders or laminopathies (3) that include dilated cardiomyopathy (DCM), muscular dystrophy, lipodystrophy and progeria, a premature ageing syndrome.

Two prevailing hypotheses have been proposed to explain the aetiology of laminopathies (4). The gene regulation model emphasizes the role of the lamina as a spatial modulator of gene expression (5). Here, laminopathies result from dysregulation of key genes. In contrast, the structural model posits that the lamina protects the nucleus from mechanical injury, with lamin dysfunction, particularly in mechanically stressed tissues like striated muscle, resulting in accumulation of nuclear damage leading to cell death (6). Accordingly, studies on cardiomyocytes harbouring laminopathy mutations have found only modest irregularities in gene expression and chromatin organization when compared with wild-type cells (7–10). On the other hand, upregulation of lamin A/C expression has been shown to protect cardiomyocytes from mechanically induced nuclear rupture (11).

Clearly, the structural hypothesis requires mechanisms for force transmission to the nucleus. Cytoskeletal proteins, including various motors, bind directly to the interphase NE (12–15), primarily via Linker of Nucleoskeleton and Cytoskeleton (LINC) complexes (16). These structures are composed of SUN (Sad1p, UNC-84) domain proteins of the INM and KASH (Klarsicht, ANC-1, Syne Homology) domain proteins of the ONM. Their C-termini physically interact within the PNS to form the LINC complex core (17,18). The N-termini of KASH proteins typically interact with the cytoskeleton (19), whereas those of SUN proteins interact with the nuclear lamina and other nuclear proteins (17,20,21). As such, SUN and KASH proteins represent links in a molecular chain that physically couples nuclear structures to the cytoskeleton and ultimately to the plasma membrane and extracellular matrix components (22).

We originally showed that deletion of the SUN domain protein, Sun1, in mice suppresses pathological consequences of *Lmna* mutations (23). However, whether its LINC complex role as an INM anchor for KASH domain proteins, a separate SUN1 function or SUN1 overexpression toxicity was responsible for the protective effect was unclear (24). Recent work employing either dominant negative SUN or KASH constructs indicates that it is indeed LINC complex disruption that suppresses a variety of *Lmna* mutations in cardiac (25), skeletal and smooth muscle (26,27). Significantly, loss of Sun2, the abundant Sun1 paralogue, did not rescue *Lmna* pathology, pointing to the specific protective contribution of SUN1 LINC complexes. These findings also indicate that SUN protein functions cannot be wholly redundant (25). Crucially, however, the KASH domain protein partner(s) of SUN1 that actually drives *Lmna* pathology remains unknown.

Nuclear pathology in laminopathies has been experimentally linked to the microtubule (MT) system, since depleting *Lmna*-mutant myotubes of kinesin represses the accumulation of nuclear damage (26). In earlier studies, we and others documented MT-mediated nuclear migration in myotubes (13–15,28,29) and identified Nesprin-1α (encoded by *Syne1*) as the NE-localized KASH protein adaptor required for this process (30,31). In this context, Nesprin-1α represents an NE-associated binding partner for Kinesin-1 (32) as well as for a variety of centrosomal proteins, including AKAP450 and pericentrin, that seed MT growth from the NE. In addition, Nesprin-1α also contains a binding site for BicD2, a regulator of cytoplasmic dynein (12). These findings led us to speculate that Nesprin-1α or another Nesprin-1 isoform might be the KASH domain protein involved in deleterious force transduction to the nucleus in striated muscle laminopathies. To test this, we disrupted the KASH domain of Nesprin-1 in mice and found that this indeed rescued the pathology associated with *Lmna* mutation. Most significantly, we observed that NE localization of MT components (including MTs themselves, centrosomal proteins and motor complexes), nuclear positioning and nuclear morphology are perturbed in striated muscle of Nesprin-1-mutant mice, and that these effects appear to be associated with attenuated nuclear force transmission. Our data support the structural model for laminopathies and suggest that these disorders could be treated by the selective disruption of Nesprin-1/Sun1 LINC complexes.

## Results

### Disruption of the Nesprin-1 KASH domain has no overt detrimental effects in wild-type mice

**Figure 1:**
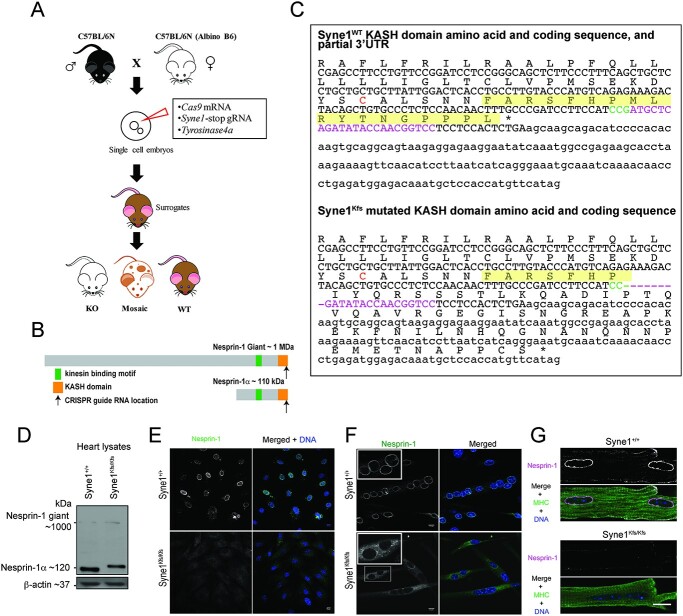
CRISPR targeting of 3′ end of *Syne1* coding sequence disrupts Nesprin-1 KASH domain in mice. (**A**) Schematic for generation of a *Syne1* mutation by microinjection of CRISPR components into mouse zygotes. Cas9 mRNA and guide RNAs targeting *Syne1* and *tyrosinase* are microinjected into mouse zygotes obtained by crossing C57/Bl6 male mice with albino C57/Bl6 female mice. Founder mice are selected from mice with white or mosaic coats resulting from CRISPR activity. (**B**) Schematic showing CRISPR guide RNA location in the cognate region of the KASH domain of Nesprin-1 Giant and α isoforms. (**C**) Wild-type and KASH mutant *Syne1* amino acid and DNA sequences, with location of guide RNA sequence in magenta and protospacer adjacent motif sequence in green. The minimal 18 amino acid SUN-binding region is highlighted in yellow and the cysteine at position −23 that forms a disulphide bond with a corresponding cysteine in the SUN domain is in orange font. (**D**) Heart tissue dissected from *Syne1^+/+^* and *Syne1^Kfs/Kfs^* mutant mice. Tissue lysates were analysed by Western blot using antibodies against Nesprin-1 and β-actin. (**E**–**G**) Immunofluorescence microscopy of fibroblasts (**E**), myotubes (**F**) and cardiomyocytes (**G**) isolated from skeletal muscle and hearts of *Syne1^+/+^* and *Syne1^Kfs/Kfs^* mice. Fibroblasts and myotubes were immunostained for Nesprin-1 (green), whereas cardiomyocytes were immunostained for Nesprin-1 (magenta), and myosin heavy chain. DNA (blue) is revealed by staining with Hoechst dye. Scale bar, 10 μm.

To determine whether Nesprin-1-containing LINC complexes drive pathology in *Lmna*-mutant mice, we derived animals deficient in NE-associated Nesprin-1 by microinjecting clustered regularly interspaced short palindromic repeats (CRISPR) components into mouse zygotes to disrupt the Nesprin-1 KASH domain. A founder animal was isolated with an 8 bp deletion in the *Syne1* terminal exon causing a frameshift (Fig. 1A and B) that is predicted to result in an extended C-terminus (by 50 amino acid residues) and loss of 11 of the 18 minimal amino acids required for SUN binding, including the critical PPPL sequence at the C-terminus (Fig. 1C) (33). The new 61-residue out-of-frame sequence has no significant similarity to any other proteins currently in the National Center for Biotechnology Information (NBCI) non-redundant protein sequences database. Notably, the transmembrane region of the KASH domain is unaffected by the frameshift. *Syne1^Kfs/Kfs^* (KASH frameshift) mice were born at normal Mendelian ratios, with no differences in body weight when compared with wild-type littermates and with no overt pathologies. Western blots of cardiac tissue show the predominant Nesprin-1α isoforms migrating slightly more slowly, consistent with the 50 residue C-terminal extension (Fig. 1D). In addition, levels of the mutant form of Nesprin-1 appeared to be marginally reduced when compared with its wild-type counterpart. The significance of this slight difference is unclear. In skeletal muscle lysates, a more complex array of Nesprin-1 isoforms was observed, with negligible differences in expression levels between wild-type and mutant samples (Supplementary Material, Fig. S1A). As expected with a mutated KASH domain, Nesprin-1 was not localized to the NEs of fibroblasts, myotubes or cardiomyocytes derived from mutant animals (Fig. 1E–G). Instead, it appeared to be largely distributed throughout a peripheral membrane-like structure, most likely the endoplasmic reticulum (ER).

### Nesprin-1/Sun1 LINC complexes are required for appropriate localization of MT cytoskeleton proteins to striated muscle NEs

We and others have examined the role of LINC complexes in recruiting MT cytoskeleton proteins to the NE in skeletal muscle cells (13–15,28–31). In particular, we showed that the LINC complex protein, Nesprin-1, functions as an ONM adaptor for both Kinesin-1 and microtubule organizing centre (MTOC) proteins, such as AKAP450 and pericentrin. Immunofluorescence microscopy of myotubes differentiated from *Syne1^Kfs/Kfs^* mouse primary myoblasts revealed that, consistent with earlier reports (30,31), disruption of the Nesprin-1 KASH domain resulted in mislocalization of MTOC components, Pericentrin (Pcnt), Akap450 and PCM1, from the myotube NE (Supplementary Material, Fig. S1C). Other NE components, including Sun1, lamin A/C and emerin, an INM protein, remained unaffected (Supplementary Material, Fig. S1B and D). In contrast to wild-type myotubes where nuclei are uniformly distributed, the *Syne1^Kfs/Kfs^* myotubes featured nuclei clustered at the cell centre (Supplementary Material, Fig. S1E and F).

**Figure 2:**
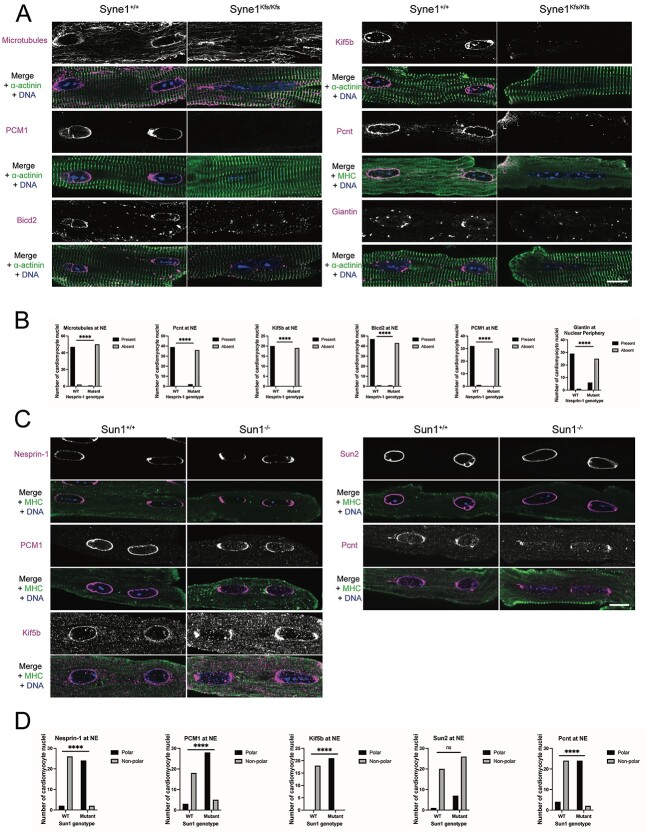
Mislocalization of microtubule cytoskeleton proteins in LINC complex mutant cardiomyocytes. Immunofluorescence microscopy of cardiomyocytes isolated from 6 to 8 weeks old mice. Cardiomyocytes isolated from *Syne1^+/+^* and *Syne1^Kfs/Kfs^* mice (**A**) immunostained for microtubules, kinesin heavy chain (Kif5b), PCM1, Pericentrin (Pcnt), Bicd2 or giantin (all in magenta) and α-actinin (green) or myosin heavy chain (MHC, green). Representative images of binucleated cardiomyocytes are shown. (**B**) Quantification of number of *Syne1^+/+^* or *Syne1^Kfs/Kfs^* cardiomyocyte nuclei with protein of interest present at (black) or absent from (grey) the nuclear envelope (NE). Fisher’s exact test was used to determine statistical significance. ^*^^*^^*^^*^P < 0.0001. (**C**) Cardiomyocytes isolated from *Sun1^+/+^* and *Sun1^−/−^* mice immunostained with antibodies against Nesprin-1, Sun2, PCM1, Pcnt (Pcnt) or kinesin heavy chain (Kif5b) (all in magenta) and myosin heavy chain (MHC, green). Representative images of binucleated cardiomyocytes are shown. In all samples, DNA (blue) is revealed by staining with Hoechst dye. Scale bar, 10 μm. (**D**) Quantification of number of *Sun1^+/+^* or *Sun1^−/−^* cardiomyocyte nuclei with protein of interest localized (polar, black) or not localized (non-polar, grey) at poles of the nuclear envelope (NE). Fisher’s exact test was used to determine statistical significance. ^*^^*^^*^^*^P < 0.0001; ns, not significant.

Although the cardiomyocyte NE has long been known to function as an MTOC (34–36), the status of MT components as well as the identity of NE-associated MT adaptors remained largely unexplored. Accordingly, we sought to determine whether Nesprin-1 was required for retention of MT cytoskeleton and MTOC proteins at the cardiomyocyte NE. In isolated adult mouse cardiomyocytes, we detected an NE pool of MTs using the YOL1/34 anti-α-tubulin monoclonal antibody (Fig. 2A and B) (37). This pool was lost in *Syne1^Kfs/Kfs^*-mutant cardiomyocytes. Similarly, the MTOC proteins Pcnt and PCM1, which are normally localized to the cardiomyocyte NE (35), were also displaced in *Syne1^Kfs/Kfs^* cardiomyocytes (Fig. 2A and B). In rat neonatal cardiomyocytes, and mouse and human myotubes and muscle fibres, the Pcnt paralogue AKAP450 (38) also relocates to the NE and is essential for the recruitment of MTs to the myotube NE (31,36,39). Surprisingly, however, in both mature and neonatal wild-type and *Syne1^Kfs/Kfs^* cardiomyocytes, Akap450 was not found at the NE. Instead, it localized exclusively to cytoplasmic foci (Supplementary Material, Fig. S2A). Akin to the MTOC proteins, Kinesin-1 heavy chain Kif5b and the dynein adaptor Bicd2 also display Nesprin-1-dependent association with the cardiomyocyte NE (Fig. 2A and B). Similarly, the Golgi protein Giantin is dependent upon Nesprin-1 for enrichment at the perinuclear region of cardiomyocytes (34).

We were also particularly curious as to the effect of lamin A/C loss on cardiomyocyte MTOC protein localization, since, as will be described below, this will affect our view of how LINC complexes might affect the progression of striated muscle laminopathies. Studies on fibroblasts derived from *Lmna^−/−^* embryos had previously suggested a partial decoupling of the single cytoplasmic MTOC from the nuclear periphery. This effect, observed as a small 1–1.5 μm shift in the centrosome away from the NE, was allied with mislocalization of emerin, an INM protein that interacts directly with A-type lamins (40). Other studies, however, have shown that, in contrast to emerin, appropriate localization of LINC complexes and their constituents to the NE is largely unaffected by A-type lamin depletion (41). This in turn is consistent with our own findings that in *Lmna*-null cardiomyocytes, MTOC components, including PCM1, are efficiently recruited to and retained at the NE (Supplementary Material, Fig. S2B).

Previously, loss of Sun1 was suggested to lead to reduction of Pcnt at the myotube NE, with the residual protein often concentrating at either end of the elongated nucleus (31). More recently, observations in Sun1-null cardiomyocytes revealed that while the bulk of Nesprin-1 was lost from the NE, a residual population was also found mislocalized to the nuclear poles (25). Even in wild-type cardiomyocytes, a partial enrichment of Pcnt at either pole of the nucleus can sometimes be observed (Supplementary Material, Fig. S2C and D). We documented similar asymmetric distributions for PCM1 and Kif5b in Sun1-null cardiomyocytes, consistent with their association with Nesprin-1α (Fig. 2C and D). Importantly, the localization of Sun2 in cardiomyocytes is largely unaffected by Sun1 depletion (Fig. 2C and D).

### Nesprin-1/Sun1 regulate nuclear morphology and positioning in striated muscle cells

**Figure 3:**
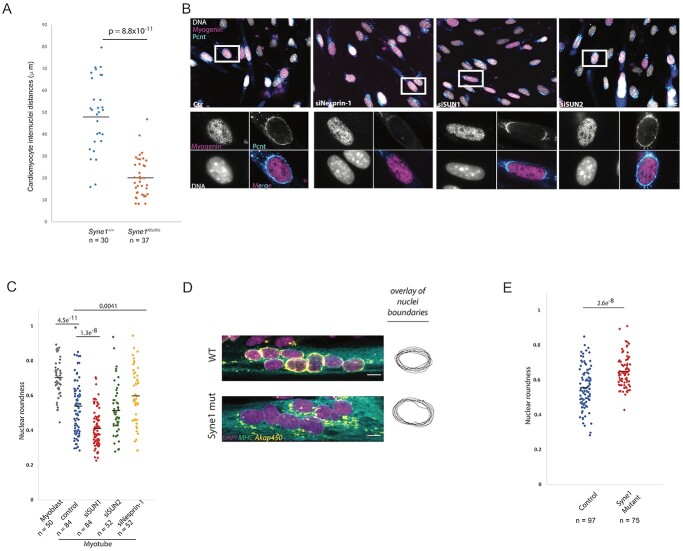
LINC complexes influence nuclear positioning, nuclear morphology and MTOC protein localization in striated muscle cells. Nuclear positioning in wild-type and *Syne1^Kfs/Kfs^* binucleate cardiomyocytes (**A**) was determined by measuring the distance between cell centroids and nuclei. *n* represents the number of binucleate cardiomyocytes assessed from at least three mice, *P*-value determined by *t*-test. (**B**) Immunofluorescence microscopy of differentiated C2C12 cells showing Pericentrin (Pcnt, cyan), myogenin (magenta) and DNA (grey) after silencing of the indicated proteins. Polarization of Pcnt staining is visible at nuclear poles along the cell axis following Sun1 depletion. NE-associated Pcnt is eliminated following Nesprin-1 depletion. Bar is 50 μm. Nuclear roundness is affected by silencing of both Sun1 and Nesprin-1 (**C**). Sun1-depleted myotubes have more elongated nuclei than control myotubes. In contrast, Nesprin-1 depletion results in increased nuclear roundness. *n* represents number of myotubes from three independent experiments, *P*-value indicated at top of graph determined by *t*-test. (**D**) Wild-type and *SYNE1* mutant immortalized human myotubes immunostained for myosin heavy chain (MHC, green) and Akap450 (yellow). DNA (magenta) was revealed by DAPI staining. Overlays of nuclear boundaries from each genotype are shown to the right of the images to highlight differences in nuclear roundness. Bar, 20 μm. (**E**) Morphometric analysis human myotube nuclei reveals increased roundness associated with *SYNE1* mutation. *n* represents number of myotubes from three independent experiments, *P*-value indicated at top of graph determined by *t*-test.

Nuclear positioning and nuclear shape depend upon forces transmitted to the nucleus from the cytoplasmic and even extracellular environments via LINC complexes (42), with the MT cytoskeleton playing a central role in many cell types (43). The Nesprin-1-dependent presence of kinesin at the cardiomyocyte NE implies that, as in myotubes, the nuclei are actively positioned in typically binucleate mouse cardiomyocytes by MT motor activity. Echoing a prior report (44), we observed that inter-nuclear distance decreased in Syne1^Kfs/Kfs^-mutant cardiomyocytes from 48.8 ± 3.0 to 20.3 ± 1.5 μm when compared with wild-type cells (Fig. 3A). Typically, wild-type cells maintained a symmetric nucleus-to-cell-centroid spacing of 26.6 ± 1.5 μm, compared with 8.7 ± 1.0 μm for Nesprin-1 mutants. The latter situation was usually manifested as pairs of nuclei positioned at the cell centre, with the individual nuclei frequently appearing to physically contact each other (Supplementary Material, Fig. S2E).

To understand how LINC complex–cytoskeleton interactions might shape nuclei, we turned to the more tractable myotube system. Nuclei in myotubes are usually more elongated than in myoblasts, presumably due to the vectorial organization of the myotube cytoskeleton, particularly MTs, and the resultant asymmetry of the forces exerted on the NE (Fig. 3B and C). However, nuclei in myotubes depleted of Nesprin-1 or harbouring a Nesprin-1 mutation linked to human muscular dystrophy (45) adopt a more rounded appearance than in wild-type cells (Fig. 3B–E). Myotube nuclei lacking Nesprin-1 are deficient in both MTOC proteins and MTs at the NE (Fig. 3B and D), suggesting decoupling of the nucleus from the MT system, resulting in a diminution in anisotropic MT forces acting to shape nuclear morphology (Fig. 3, Supplementary Material, Fig. S2).

Nesprin-1 is anchored at the NE by SUN proteins. Although loss of Sun2 had little discernible effect on nuclear morphology, the absence of Sun1 results in nuclear elongation (Fig. 3B and C), the reverse of what happens following Nesprin-1 depletion (Fig. 3B–E). Why would loss of Sun1 versus Nesprin-1 differentially shape nuclear morphology? A clue may lie in work showing that LINC complexes engaged with the MT system contain predominantly Sun1 (46,47). In Sun1, but not control or Sun2-depleted cells, Nesprin-1 and Pcnt are indeed largely, but not completely, lost from the nuclear surface, with the residual protein populations concentrated at the nuclear poles, similar to what occurs in Sun1-deficient cardiomyocytes (Supplementary Material, Fig. S2F, Fig. 2B). We would suggest that without Sun1, MT-engaged Nesprin-1 is bound, albeit less effectively, by Sun2. In Sun1-null myotubes, longitudinal MT arrays would direct Nesprin-1 and associated proteins (Pcnt, Kinesin, etc.), weakly anchored by Sun2, towards either pole of the nucleus. Consistent with this notion, more elongated nuclei appear to have a more polarized distribution of NE-associated Pcnt and Nesprin-1, likely correlated with elevated MT forces along the nuclear long axis (Supplementary Material, Fig. S2G). If this suggestion is true, then eliminating both Sun1 and Sun2 should result in complete loss of Nesprin-1 and MT components from the NE, and indeed this is exactly what happens (31).

### Nesprin-1 mutation suppresses pathology in Lmna-mutant mice

**Figure 4:**
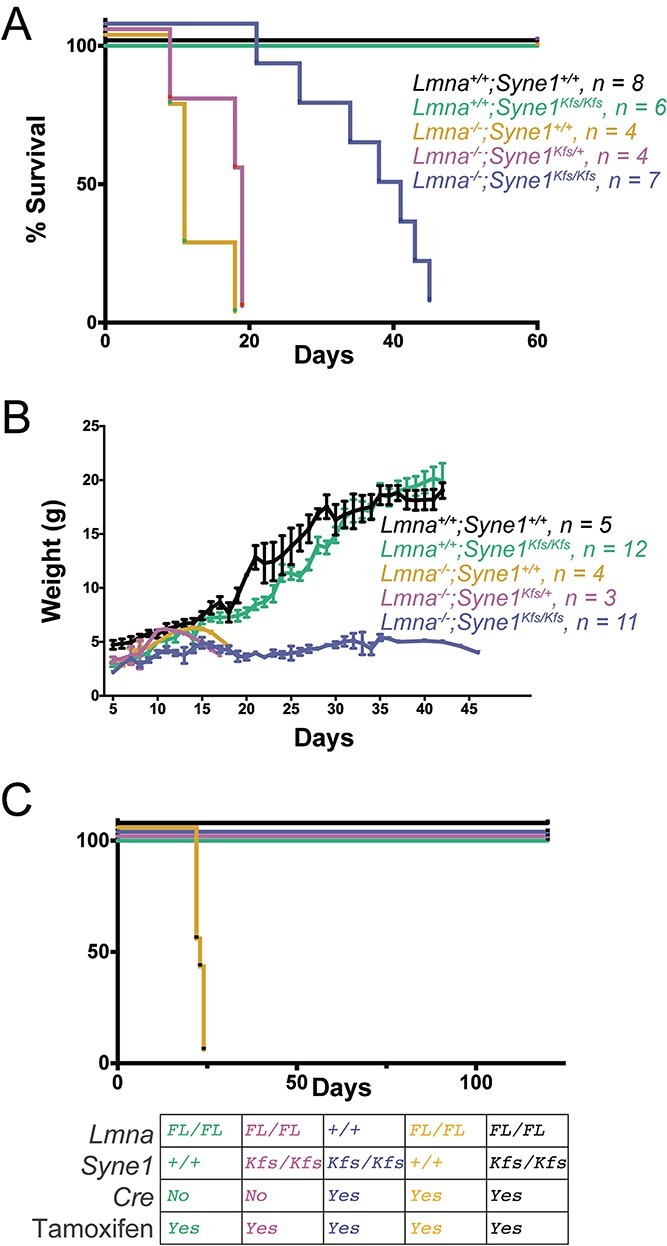
Disruption of Nesprin-1 KASH domain suppresses mortality resulting from loss of lamin A/C. (**A** and **B**)  *Lmna^+/−^/Syne1^+/Kfs^* mice were intercrossed to obtain *Lmna^+/+^/Syne1^+/+^*, *Lmna^+/+^/Syne1^Kfs/Kfs^*, *Lmna^−/−^/Syne1^+/+^, Lmna^−/−^;Syne1^Kfs/Kfs^* mice and their survival (A) and weight (B) were monitored for up to 2 months. (**C**) Mice were obtained by appropriate crosses to generate the following genotypes: Lmna^FL/FL^;Tg(Myh6-cre/Esr1^*^);Syne1^+/+^, Lmna^FL/FL^;Tg(Myh6-cre/Esr1^*^);Syne1^Kfs/Kfs^, Lmna^+/+^;Tg(Myh6-cre/Esr1^*^);Syne1^Kfs/Kfs^ and *Lmna^FL/FL^; Syne1^+/+^, Lmna^FL/FL^; Syne1^Kfs/Kfs^*. Genotypes are colour coded and indicated in the table below the graph. Following treatment with tamoxifen, lifespan was monitored. It is clear from these results that *Syne1^Kfs^* extends lifespan in mice with *Lmna* deletion.

Loss of functional A-type lamins is associated with striated muscle diseases that, in humans, include Emery–Dreifuss muscular dystrophy and DCM. *Lmna*-null mice represent a viable model for various forms of human *LMNA*-linked muscular dystrophy (48). Although the aetiology of the muscle pathology in these mice is uncertain, there is a growing appreciation that it is associated with increased mechanical fragility of muscle nuclei. For example, the MT motor protein, kinesin, was proposed to promote DNA damage in mechanically compromised *Lmna*-mutant myotube nuclei as a direct consequence of its role in MT-dependent nuclear positioning during myogenesis (26). This damage appeared to be LINC complex mediated, although it was unclear whether it was the LINC complex associated or cytoplasmic pools of kinesin that was responsible. We have now identified an absolute requirement for Nesprin-1, specifically the short Nesprin-1α isoform, in recruiting kinesin and other MT elements to the striated muscle NE. Nesprin-1 is thus most likely the missing link between kinesin and nuclear damage in *Lmna*-mutant myotubes. If so, then disruption of Nesprin-1 function should ameliorate the effects of *Lmna* mutation. To test this, we examined mice harbouring combinations of *Lmna*-null and *Syne1^Kfs^* alleles. We used recently described *Lmna* global null mice (*Lmna^−/−^*) derived by crossing *Lmna* ‘floxed’ mice to a zygotic Cre driver (25). These mice die within 3 weeks of birth (Fig. 4A). However, the life expectancy of *Lmna^−/−^/Syne1^Kfs/Kfs^* mice was more than doubled to 6–7 weeks. Evidently loss of functional Nesprin-1 suppresses the effects of *Lmna* deletion. This lifespan extension in *Lmna*-null mice mirrors that produced by homozygous deletion of Sun1, although in both situations the double mutant mice still display reduced body weight throughout their extended lifespan (Fig. 4B).

Is disruption of the Nesprin-1 paralogue Nesprin-2 similarly beneficial? Mice with a mutation deleting part of exon 102 and all of exons 103–104 of *Syne2* were generated by conventional gene targeting (*Syne2^Cdel/Cdel^*, Supplementary Material, Fig. S3A). As expected, homozygous animals displayed no overt phenotype (49), although immunostaining confirmed loss of Nesprin-2 protein at the NE in *Syne2^Cdel/Cdel^* fibroblasts (Supplementary Material, Fig. S3B). Western blot analysis of fibroblast lysates revealed a ~100 kDa Nesprin-2 isoform in both wild-type and *Syne2^Cdel/Cdel^*, suggesting a mutant form of Nesprin-2 is still expressed in *Syne2^Cdel/Cdel^* mice (Supplementary Material, Fig. S3C). As previously reported, double homozygous Syne1/2 mutant (*Syne1^Kfs/Kfs^; Syne2^Cdel/Cdel^*) mice exhibited embryonic or perinatal lethality, confirming loss of essential Nesprin-2 function in *Syne2^Cdel/Cdel^* animals (Supplementary Material, Fig. S3D). When crossed with *Lmna* global null mice, the *Syne2* mutation, in contrast to the *Syne1* mutation, failed to suppress the effects of *Lmna* deletion (Supplementary Material, Fig. S3E and F).

**Figure 5:**
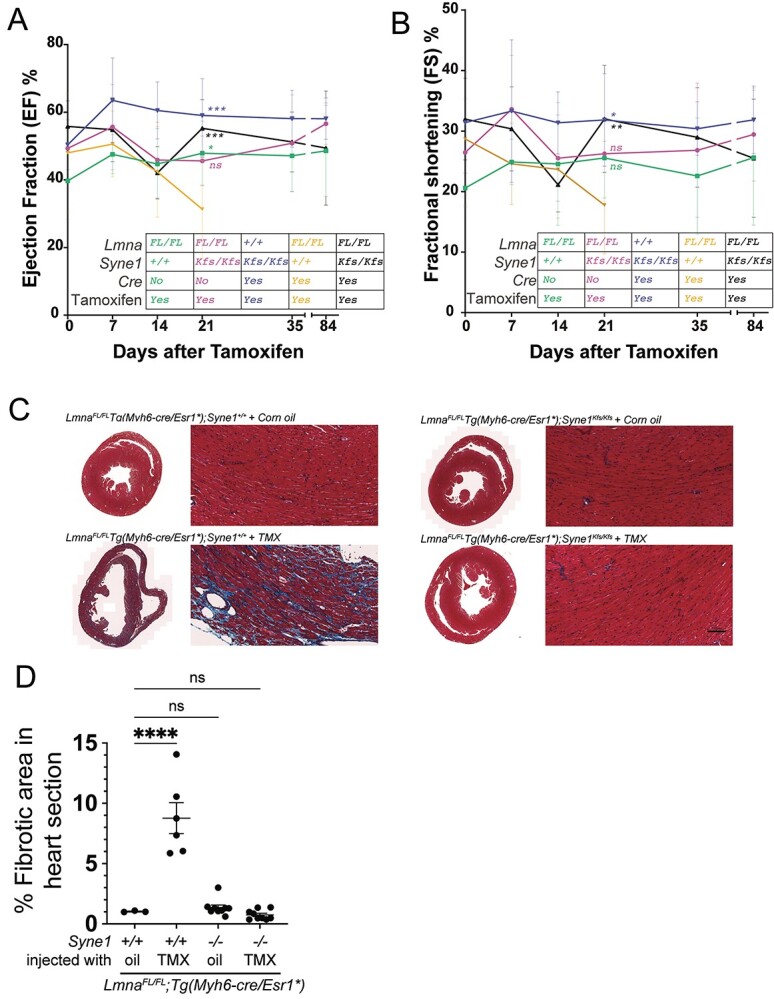
Disruption of Nesprin-1 KASH domain suppresses cardiac pathology resulting from loss of lamin A/C in cardiomyocytes. Mice were obtained by appropriate crosses to generate the following genotypes: Lmna^FL/FL^;Tg(Myh6-cre/Esr1^*^);Syne1^+/+^, Lmna^FL/FL^;Tg(Myh6-cre/Esr1^*^);Syne1^Kfs/Kfs^, Lmna^+/+^;Tg(Myh6-cre/Esr1^*^);Syne1^Kfs/Kfs^ and *Lmna^FL/FL^; Syne1^+/+^, Lmna^FL/FL^; Syne1^Kfs/Kfs^*. Following treatment with tamoxifen, echocardiography analysis was conducted to determine ejection fraction (**A**) and fractional shortening (**B**). Genotypes are colour coded and indicated in the table in each graph. Statistical significance was determined by comparing each genotype to every other one using ordinary one-way ANOVA and Tukey’s multiple comparisons test. *P*-values were annotated on the graphs for each genotype relative only to Lmna^FL/FL^;Tg(Myh6-cre/Esr1^*^);Syne1^+/+^ mice. ^*^^*^^*^P < 0.001; ^*^^*^P < 0.01; ^*^P < 0.05; ns, not significant. Transverse sections of heart tissue (**C**) were obtained and subjected to Masson’s trichrome staining for mice with the following genotypes and treatments: Lmna^FL/FL^;Tg(Myh6-cre/Esr1^*^);Syne1^+/+^ treated with corn oil, Lmna^FL/FL^;Tg(Myh6-cre/Esr1^*^);Syne1^*+/*+^ treated with tamoxifen (TMX), Lmna^FL/FL^;Tg(Myh6-cre/Esr1^*^);Syne1^Kfs/Kfs^ treated with corn oil and Lmna^FL/FL^;Tg(Myh6-cre/Esr1^*^);Syne1^Kfs/Kfs^ treated with tamoxifen (TMX). (**D**) The area of fibrosis in tissue sections from each genotype was quantified using Fiji software. Statistical significance was determined by comparing each genotype to Lmna^FL/FL^;Tg(Myh6-cre/Esr1^*^);Syne1^+/+^ mice treated with corn oil using ordinary one-way ANOVA and Dunnet’s multiple comparisons test. ^*^^*^^*^P < 0.001; ^*^^*^P < 0.01; ^*^P < 0.05; ns, not significant. It is clear from these results that *Syne1^Kfs^* effectively suppresses the deleterious effects of *Lmna* deletion in cardiomyocytes.

Previously, we used cardiac-specific loss of *Lmna* in mice to model *LMNA* DCM, the second most common genetic cause of DCM (50). Normally, these mice die of DCM a month after tamoxifen-induced *Lmna* deletion (25). We wondered if the suppressor effect of Nesprin-1 extended to *LMNA* DCM. We found that *Lmna* cardiac-mutant mice with the *Syne1^Kfs/Kfs^* mutation lived at least 4 months, the time-point at which mice were sacrificed (Fig. 4C). Cardiac physiology was monitored both before and after tamoxifen treatment using echocardiography (Fig. 5A and B). Mice with the cardiac-specific *Lmna* mutation alone exhibited a decline in ejection fraction (EF) and fractional shortening (FS) 3–4 weeks after tamoxifen induction, whereas *Lmna;Syne1* double mutant mice were indistinguishable from wild-type or *Syne1* mutant animals. When histological sections of hearts from the various wild-type and mutant mice were examined, only those harbouring the *Lmna* mutation alone exhibited characteristics of DCM such as thinner ventricular walls and extensive fibrosis (Fig. 5C and D). These features were virtually eliminated in tamoxifen-treated Lmna^FL/FL^; Tg(Myh6-cre/Esr1^*^); Syne1 ^Kfs/Kfs^ mice. Thus, similar to loss of Sun1 (25), disrupting the KASH domain of Nesprin-1 preserves almost complete function in *Lmna-*mutant cardiomyocytes.

Nesprin-1/Sun1 LINC complexes normally recruit kinesin and other MT elements to striated muscle NEs to regulate nuclear positioning and morphology. To exclude the possibility that *Lmna* mutation might influence these *Syne1^Kfs/Kfs^* phenotypes, we examined *Syne1^Kfs/Kfs^* cardiomyocytes where the *Lmna* gene was either intact or mutated. As expected, MT elements were lost from the NE, and inter-nuclear distance was similarly reduced, in both *Lmna* wild-type and *Lmna*-mutant cardiomyocytes only where Nesprin-1 was mutated (Supplementary Material, Fig. S4).

## Discussion

We have shown that disrupting the KASH domain of Nesprin-1 results in mislocalization of several MT cytoskeleton proteins from the NE of striated muscle cells, but has negligible effects on mouse physiology. However, mutation of the Nesprin-1 KASH domain and concomitant displacement of MT cytoskeleton proteins from the NE is beneficial in the context of *Lmna* mutations. This suggests laminopathy phenotypes result from MT cytoskeleton activity at the NE.

The lack of any gross phenotype in our Nesprin-1 mice is mirrored in another line where the terminal coding exon of Nesprin-1 was partially replaced (49), resulting in the deletion of SUN-interacting sequences. This particular mutation removes most of the KASH domain at the Nesprin-1 C-terminus, whereas our Kfs mutation introduces an additional 50 residues. The key point here, however, is that in both cases the C-terminal tetrapeptide (PPPL, single letter code), which is crucial for the SUN–KASH interaction is eliminated. In contrast, cardiac and muscle phenotypes were observed in two additional Nesprin-1-mutant strains where the mutations likely impaired other aspects of Nesprin-1 function (51,52). For example, in the mice described by Puckelwartz *et al*. (51), the last 100 amino acids (as opposed to 11 in *Syne1^Kfs/Kfs^*) are replaced by 61 amino acids of novel sequence, and unlike in *Syne1^Kfs/Kfs^* mice, the KASH transmembrane region is lost. This notion is further supported by Nesprin-1 separation of function mutations targeting the N-terminal actin-binding domain versus the Nesprin-1α-specific exon, which yield either no phenotype or cardiac and muscle phenotypes, respectively (53). Incomplete penetrance of perinatal mortality was also observed in some of these lines, perhaps due to mixed genetic backgrounds. By generating our mutation in inbred C57/B6 zygotes, we removed complicating effects of variable modifier mutations (Fig. 1). Human mutations in *SYNE1* associated with cardiac, muscle or neurological disorders have also been reported (54–58). The specific *SYNE1* mutation and genetic background will likely influence disease penetrance in humans. However, merely disrupting the Nesprin-1 KASH domain in a limited way may be largely benign (Fig. 1).

We know from work presented here (Figs 2 and 3) and from previous studies that LINC complexes represent the primary anchors for MTOC and other MT components at the NEs of striated muscle cells. Here, we found that Nesprin-1 specifically is required for NE anchoring of many MT components, except Akap450, which appears distributed in the cytoplasm of both wild-type and Nesprin-1-mutant cardiomyocytes. Thus, in contrast to the situation in skeletal muscle, Nesprin-1 does not contribute to the localization of AKAP450 in mouse cardiomyocytes. This difference in AKAP450 distribution might reflect alternative splicing of its pre-messenger RNA (mRNA) or differences in post-translational modifications in myotubes or rat cardiomyocytes. Whatever the reason, the take-home message here is that in adult cardiomyocytes, AKAP450 can have no significant role in the nucleation of MTs at the NE. In contrast, recruitment of MTs, MTOC and cytoplasmic dynein to the NE by Nesprin-1 likely contributes to NE localization of Golgi proteins like Giantin, akin to the perinuclear localization of the Golgi apparatus as a whole in the majority of cell types (59).

Our finding that SUN1 loss results in polarization of MT components at the NE (Figs 2 and 3) supports the view that while the two SUN proteins share significant functional overlap (60), they are not fully redundant and that SUN1 is primarily responsible for MT-dependent force transduction. In contrast, given that PCM1 still localizes to the cardiomyocyte NE in *Lmna-*null cardiomyocytes, lamin A/C and its interactor, emerin, apparently play little or no part in the reorganization of the MTOC in cardiomyocytes.

Our findings also indicate that Sun1 is an important determinant of Nesprin-1α and MT component NE localization in cardiomyocytes. Interestingly, a recent report by Heffler *et al*. revealed that MTs and desmin, but not actin, exert countervailing forces to shape the cardiomyocyte NE in a LINC complex-dependent manner (61). Here, we establish that Nesprin-1, in concert mainly with Sun1, acts to recruit MTs to position and shape nuclei in striated muscle by co-opting the NE as a primary MTOC. Clearly, Nesprin-1 with its Sun1 partner is key to the generation and dissemination of MT-dependent forces in both cardiac and skeletal muscle.

We determined that the Nesprin-1, but not the Nesprin-2, mutation we generated suppresses pathology associated with *Lmna* mutations in two mouse models (Figs 4 and 5). Interestingly, the rescue effect of the Nesprin-1 mutation was more apparent in the cardiac-specific *Lmna* mutation than in the global *Lmna* null. In the absence of additional genetic crosses, we can only speculate that the global *Lmna*-null mice suffer skeletal muscle tissue defects that are much more deleterious to the health of the organism than the cardiac-specific *Lmna* deletion, potentially because muscle tissue experiences more mechanical stress than the heart. Although it is possible that other mutations to Nesprin-2 would have different effects, the lifespan increase of *Lmna-*mutant mice following either Nesprin-1 KASH disruption (Figs 4 and 5) or Sun1 (but not Sun2) loss (25) suggests that LINC complexes containing Sun1 and Nesprin-1 are involved in *Lmna*-linked pathology, whereas the role of Nesprin-2 in such pathology remains to be fully elucidated.

**Figure 6:**
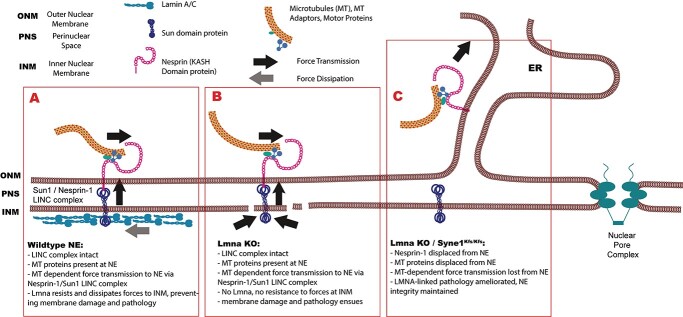
Model for the coupling of nuclei to the microtubule system: how LINC complex depletion might protect nuclei deficient in A-type lamins from mechanical damage. LINC complexes containing SUN1 and Nesprin-1 transmit forces generated by the microtubule (MT) system across the NE to nucleoplasmic components, including the nuclear lamina (**A**). Since components of the microtuble cytoskeleton apply forces directly to the KASH domain proteins of the LINC complex, the arrows suggest how forces might be dissipated and transmitted relative to the nuclear membranes. The nuclear lamina resists forces applied to the NE to the via LINC complexes, thereby maintaining NE and nuclear integrity (A). In the absence of A-type lamins, the NE is more prone to deformation. Consequently, forces applied to LINC complexes in these structurally compromised NE by MTs and MT motor proteins may lead to rupturing of the nuclear membranes (**B**). In *Syne1^Kfs/Kfs^* cardiomyocytes, Nesprin-1 cannot engage with Sun1 and is therefore lost to the sarcoplasmic reticulum (**C**). In this way, the NE is uncoupled from the MT system and consequently is protected from deleterious MT-generated forces.

Based on the findings described above, we propose a model in which force-generating activities that are coupled to Nesprin-1/Sun1 LINC complexes promote the disruption of structurally compromised nuclei in *Lmna*-mutant striated muscle cells (Fig. 6). In this scheme forces transmitted to the NE by the MT system and LINC complexes would be resisted by the nuclear lamina, particularly the A-type lamins (Fig. 6A). In situations where functional A-types lamins are absent, MT generated forces result in deformation of the nucleus and ultimately rupture of the nuclear membranes, culminating in irreversible cell and tissue damage (Fig. 6B). By displacing Nesprin-1 from LINC complexes, either through KASH-domain mutation or through expression of dominant negative SUN1, MTs are uncoupled from the NE, resulting in decreased nuclear and NE perturbation and preservation of cell viability (Fig. 6C). Although we have not directly examined nuclear damage at the cellular level, the extent of tissue damage in mouse models of *Lmna*-linked muscle disease has been extensively documented. Consistent with prior suggestions of MT involvement in *Lmna*-linked DCM (62), mislocalization of Nesprin-1 likely rescues *Lmna* pathology by dispersing MT-mediated forces away from nuclei to the sarcoplasmic reticulum (63). In this study, we used nuclear deformation as a qualitative readout for force transmission to the NE. Although we have indicated possible directions of force transmission and dissipation in Figure 6 relative to the nuclear membranes, these are merely suggestions that would need to be experimentally tested. Quantitative analysis in primary cell cultures and possibly tissues would likely require engineering of Nesprin tension sensors (64–66) into mice. Such analyses clearly lie beyond the scope of the current study but could provide important insight into the role of LINC complexes in diverse cell types.

Given the benign nature of our specific KASH mutation, we suggest that the SUN-binding function of Nesprin-1, unlike kinesin, represents a safe therapeutic target. *In vivo* CRISPR-based therapeutics (67) that mutate the terminal exon of *SYNE1* could be used to treat striated muscle laminopathies. The safety and efficacy of such an approach can now be examined in appropriate animal disease models.

## Materials and Methods

### Generation of transgenic mice and mouse genetics

Mouse (C57Bl6/J and 129Sv/J) strains were maintained at the A^*^STAR Biological Resource Centre (BRC) facility and the NUS Animal Facility on a 12 h light/dark cycle in ventilated animal barrier facilities with the temperature set to 21 ± 1°C, humidity at 55–70% and with food and water provide *ad libitum*, in accordance with all ethical regulations and approval of the Institutional Animal Care and Use Committees, for the A^*^STAR BRC.

The cardiac-specific, tamoxifen-regulated Cre allele (*α-MHC-MerCreMer*, Tg(Myh6-cre/Esr1^*^)1Jmk, JAX stock 005657) has been described (68). The *Lmna* conditional allele and the *Lmna* global null, *Lmna* cardiac conditional null and *Sun1*-null mice have also been described (25,69,70). To generate the *Syne2* mouse line, an Internal Ribosome Entry Site (IRES)–β-gal neomycin selectable cassette (PgkNeo) flanked by loxP sites was inserted into the *Syne2* gene. The last 74 nucleotides of exon 102 and all of exons 103–104 were replaced by the targeting construct, resulting in a premature stop codon 41 nucleotides from the 5′ end of the targeting construct insertion site. The targeting vector was linearized and electroporated into Bruce4 embryonic stem (ES) cells that were largely of C57BL/6 origin. Clones selected with neomycin were picked, expanded and screened for recombination. ES cells were injected into albino C57BL/6^cBrd/cBrd/Cr^ blastocysts, and chimeras were bred to produce germline offspring (71). The neomycin cassette was subsequently removed by crossing with mice harbouring the ZP3-Cre allele (Tg(Zp3-cre)93Knw, JAX stock 003651), which deletes floxed alleles in the female germline. To induce loxP recombination with cardiac Cre driver, mice were injected once with 40 mg/kg of Tamoxifen (Sigma) dissolved in corn oil (Sigma).

For generating CRISPR-modified mice, 3–4-week-old C57BL/6 N females were superovulated with Pregnant Mare Serum gonadotropin (Calbiochem, 36722, 5 IU/ml); 48 h later, the females were injected with human chorionic gonadotropin (Sigma, CG10, 5 IU/ml) and mated with C57BL6 males. The following day, fertilized 0.5dpc embryos were collected from the oviducts. Cas9 mRNA (Sigma, CAS9MRNA, 100 ng/ul), Tyrosinase4a gRNA (50 ng/ul) and gene-specific gRNA (50 ng/ul) were co-injected into the cytoplasm of the embryos in M2 medium (EmbryoMax^®^ Sigma) using a Nikon microinjection system. The injected zygotes were cultured in potassium simplex optimization medium (KSOM) with amino acids (EmbryoMax^®^ Sigma) in an incubator maintained at 37°C with 5% CO_2_ and 5% O_2_ for 2 h before implanting into 0.5dpc pseudopregnant C3H-ICR females.

*Lmna^−/−^;Syne1^Kfs/Kfs^* mice were obtained by crossing *Lmna^+/−^* and *Syne1^+/Kfs^* mice and intercrossing *Lmna^+/−^;Syne1^+/Kfs^* mice. To obtain Lmna^FL/FL^;Tg(Myh6-cre/Esr1^*^) mice, *Lmna^FL/+^* and Tg(Myh6-cre/Esr1^*^) mice were crossed, and Lmna^FL/+^;Tg(Myh6-cre/Esr1^*^) and *Lmna^FL/+^* offspring were then intercrossed. Lmna^FL/FL^;Tg(Myh6-cre/Esr1^*^) mice were crossed with *Syne1^+/Kfs^* or *Syne1^Kfs/Kfs^* to obtain Lmna^FL/+^;Tg(Myh6-cre/Esr1^*^);Syne1^+/Kfs^ mice, which were then inter-crossed to obtain Lmna^FL/FL^;Tg(Myh6-cre/Esr1^*^) mice on *Syne1^+/+^* or *Syne1^+/Kfs^ or Syne1^Kfs/Kfs^* backgrounds. *Syne1^+/Kfs^* or *Syne1^Kfs/Kfs^* mice were crossed with Syne2^+/Cdel^ or Syne2^Cdel/Cdel^ mice to obtain mice heterozygous for mutant Syne1 and Syne2 alleles, which were intercrossed to assess early lethality of double mutant mice.

CRISPR-modified mutant mice were genotyped by polymerase chain reaction (PCR) followed by gel electrophoresis using a high-resolution agarose (2% MetaPhor agarose, Lonza). Primers for genotyping *Syne1^Kfs/Kfs^* mice were as follows: N1C F2 5′-TGCTCCTGCTGCTGCTTATT-3′ and N1C-Astop R2 5′- ACATGGTGGAGCATTTGTCTCC -3′. *Syne2* mice were PCR genotyped with the following primers using conventional agarose gel electrophoresis: Nes2 WT intron102 Fwd #1 5′- TGGGCAGCAGCCATGTGAAG-3′, Nes2 WT exon103 Rev #2 5′- TCGTTCGTGAATCTGCCGCTG-3′, Nes2 LacZ Fwd #1 5′- CCGGTCGCTACCATTACCAGTTG-3′, Nes2 intron104 Rev #1 5′-GCAGAGGTGTGTCTGTTCCTGG-3′.

### Cell culture

To isolate myoblasts, limbs were obtained from euthanized mice and muscles were dissected free from bone. Tissue digestion was performed by incubating the muscle tissues in enzyme solution consisting of equal volumes of dispase II (Roche, cat. 04942078001) at a concentration of 2.4 U/ml and 1% collagenase II (GIBCO^®^ Invitrogen, cat 17101-015) in a 37°C water bath for 30 min, with periodic mixing at 10 min intervals. After 30 min, enzyme solution was neutralized in D10 medium [Dulbeco’s Modified Eagle Medium (DMEM) with 10% fetal bovine serum (FBS)]. The mixture was then filtered through 70 μm (BD Falcon^TM^, cat 352350) and 40 μm sterile filters (BD Falcon™, cat 352340). The suspension was then centrifuged, supernatant removed and cells resuspended in F10 medium (GIBCO^®^ Invitrogen, cat. 11550043) supplemented with 20% fetal bovine serum and 10 μg/ml bFGF (GIBCO^®^, cat PHG0264), and plated in 100 mm petri dishes. Mouse adult fibroblasts (MAFs) were allowed to settle for 2–3 h before collecting the supernatant (with floating myoblasts) and replated into 60 mm dishes coated with 0.15% gelatin (Sigma, cat G1393). D10 medium was added to the 100 mm plates with MAFs. To terminally differentiate myoblasts to myotubes, the media was changed to DMEM supplemented with 2% horse serum (Thermo Fisher Scientific GIBCO^®^, cat 16050122).

Myoblasts from a healthy control or from a congenital muscular dystrophy patient carrying a homozygous nonsense mutation within the SYNE1 gene (nucleotide 23560 G > T) were immortalized by Kamel Mamchaoui and Vincent Mouly (Center for Research in Myology, Paris, France) via transduction with retrovirus vectors expressing hTERT and Cdk4 as described previously (45,72). Human myoblasts were maintained in growth medium containing DMEM with GlutaMAX and DMEM 199 medium (4:1 ratio), supplemented with 20% FBS, 25 mg/ml bovine fetuin, 5 ng/ml recombinant human epidermal growth factor (EGF), 0.5 ng/ml recombinant human basic fibroblast growth factor (bFGF), 5 mg/ml recombinant human insulin, 0.2 mg/ml dexamethasone and 0.1% gentamicin. For immunofluorescence staining, human myoblasts were seeded on coverslips coated with Matrigel diluted 1:100 in DMEM, grown to 90% confluence and differentiated in Iscove’s Modified Dulbecco’s Medium with GlutaMAX, 2% horse serum and 0.1% gentamicin.

### Small interfering RNA (siRNA)/DNA transfection

C2C12 cells were transfected at ~30% confluency with siRNA (20 or 50 nM final concentration) by addition of transfection complexes pre-formed for 20 min, containing 0.3 ml Lipofectamine RNAiMAX per pmol of siRNA in Opti-MEM medium. The following siRNAs were used: siRNA non-targeting control UUCUCCGAAGCUGUCACGUtt, mouse Nesprin-1 CCAUCGAGUCUCACAUCAAtt, mouse Sun1 GGCUAUUGAUUCGCACAUUtt, mouse Sun2 CUCUCAGGAUGAUAACGAUtt. Efficacy of protein depletion for each of these siRNAs was characterized previously (31).

### Cardiomyocyte isolation

A Langendorff-free method was used to isolate cardiomyocytes (73). Mice were first anaesthetized intraperitoneally with ketamine/xylazine cocktail (150 mg/kg ketamine, 10 mg/kg xylazine). The abdominal cavity was opened, and the descending aorta and inferior vena cava were cut. The heart was flushed by injecting 7 ml of EDTA buffer [130 mM NaCl, 5 mM KCl, 0.5 mM NaH_2_PO_4_, 10 mM hydroxyethylpiperazine ethane sulfonic acid (HEPES), 10 mM glucose, 10 mM BDM (2,3-butanedione monoxime), 10 mM taurine, 5 mM ethylenediamine tetraacetic acid (EDTA) in 18.2 MΩ.cm H_2_O, adjusted to pH 7.8 and sterile filtered] into the right ventricle. The heart was removed by clamping behind the heart with Reynolds forceps and submerged in a 60 mm dish of EDTA buffer. The ascending aorta was clamped using Lahey forceps and the heart was injected with 10 ml of EDTA buffer through the apex of the left ventricle. The heart was transferred to a 60 mm dish of perfusion buffer (130 mM NaCl, 5 mM KCl, 0.5 mM NaH_2_PO_4_, 10 mM HEPES, 10 mM glucose, 10 mM BDM, 10 mM taurine, 1 mM MgCl_2_ in 18.2 MΩ.cm H_2_O, adjusted to pH 7.8 and sterile filtered) and 3 ml of perfusion buffer was injected through the same opening on the left ventricle; 30–50 ml of collagenase buffer (0.5 mg/ml Collagenase 2 and Collagenase 4, 0.05 mg/ml Protease XIV) was then injected into the left ventricle until digestion was apparent. The clamp was removed, and the digested heart was gently pulled apart into smaller pieces using forceps and gentle pipetting. 5 ml of Stop buffer (perfusion buffer with 5% sterile FBS made fresh on the day of isolation) was added to stop the enzymatic activity of collagenase. The cell suspension was passed through a 100 μm filter and the myocytes underwent four sequential rounds of gravity settling, including three rounds using calcium reintroduction buffers (combination of perfusion buffer with culture media in 3:1, 1:1, 1:3 ratios) to gradually restore physiological calcium concentration levels. The cells were resuspended in pre-warmed plating medium (M199, 5% FBS, 10 mM BDM, 100 U/ml penicillin, 100 μg/ml streptomycin) and plated on glass coverslips pre-coated with laminin (5 μg/ml). The plating medium was exchanged for culture media [M199, 0.1% bovine serum albumin, 1% ITS (1.0 mg/ml recombinant human insulin, 0.55 mg/ml human transferrin, 0.5 μg/ml sodium selenite at 100× concentration), 10 mM BDM, 1% CD lipid; chemically defined lipid concentrate (Gibco; Cat. 11905031), 100 U/ml penicillin, 100 μg/ml streptomycin, sterile filtered and protected from light] after 1 h and every 48 h thereafter.

### Molecular biology

pX330-U6-Chimeric_BB-CBh-hSpCas9 was a gift from Feng Zhang (Addgene plasmid # 42230; http://n2t.net/addgene:42230; RRID:Addgene_42230). The 20 nucleotide *Syne1* single-guide RNA sequences were designed with the help of CRISPR Design Tool (crispr.genome-engineering.org). Complimentary oligonucleotides containing the gRNA target sequences were annealed and cloned into the Bbsl site of pX330 and sequenced to verify correct insertion of the target sequences. Guide RNA sequences were as follows: 5′-CCGTTGGTATATCTGAGCAT-3′ for *Syne1*, 5′-GGTTATGGCCGATAGGTGCAT-3′ for Tyrosinase4a.

For *in vitro* transcription, PCR was performed to generate the appropriate transcription templates using a common reverse primer (AAAAGCACCGACTCGGTGCC-3′) and gRNA-specific forward primers that encoded the T7 promoter sequence as follows:

*Syne1*: 5′-TTAATACGACTCACTATAGCCGTTGGTATATCTGAGCAT-3′

*Tyrosinase4a*: 5′-TTAATACGACTCACTATAGGTTATGGCCGATAGGTGCAT-3’

The gRNA PCR products were then subjected to agarose gel electrophoresis (1.5% agarose) to confirm successful PCR, gel purified and used as templates for *in vitro* transcription using the MEGAshortscript T7 kit (Life Technologies). The gRNAs were purified using MEGAclear kit (Life Technologies) and eluted in RNase-free water. A sample of purified gRNAs was then subjected to agarose gel electrophoresis for quality checks before injecting into zygotes.

To determine sequence of CRISPR-induced mutations, PCR products from mouse tail DNA were subjected to TOPO cloning (Zero BluntTM TOPOTM PCR Cloning Kit, 450245, Thermo Fisher Scientific). Plasmid DNA from at least 10 bacterial colonies was isolated using a mini-prep kit (QIAGEN, QIAprepSpin, Miniprep Kit) and sent for Sanger sequencing.

To obtain DNA for PCR genotyping, mouse tails were clipped and each placed in a 1.5 ml Eppendorf tube; 80 μl of lysis buffer (25 mM NaOH, 0.2 mM EDTA, pH 12) was dispensed into the tube and heated at 95°C for 60 min. After heating, the buffer was neutralized with an equal volume of 40 mM Tris–HCl, pH 5. For certain applications, DNA was extracted and purified from mouse tails using DNeasy Blood and Tissue Kit (QIAGEN).

### Antibodies

Refer to Table 1 for list of antibodies used in this study.

**Table 1:** List of antibodies used in this study

Antibodies	Isotype	Source	Cat no.
Mouse monoclonal [EA-53] anti-Sarcomeric Alpha Actinin	IgG1	abcam	ab9465
MF20 (myosin heavy chain)	IgG2b	Developmental Studies Hybridoma Bank	N/A
Rabbit polyclonal anti-Pcm1 (H262)	IgG	Santa Cruz Biotechnology	sc-67204
Rabbit polyclonal anti-Pcnt	IgG	abcam	ab4448
Rabbit monoclonal [EPR10276(B)] anti-KIF5B	IgG	abcam	ab167429
Rabbit polyclonal anti-Giantin	IgG	abcam	ab24586 (discontinued)
Rabbit polyclonal anti-Bicd2	IgG	Sigma-Aldrich	HPA023013
Mouse monoclonal Anti-p150	IgG1	BD Biosciences	610474
Mouse monoclonal anti-Nesprin-1, clone 9F10	IgG2b	Gimpel *et al*.^31^	N/A
Mouse monoclonal anti-Nesprin-1, clone MANNES1E	IgG1	Glenn E. Morris	N/A
Rabbit monoclonal anti-Nesprin-1, EPR14196	IgG	abcam	ab192234
Mouse monoclonal anti-Nesprin-2, clone F-11 (epitope is situated in spectrin repeats 54–55 of Nesprin-2 giant.)	IgG1	Santa Cruz Biotechnology	sc-398616
Mouse monoclonal anti-GM130	IgG1	BD Biosciences	610822
Rat alpha Tubulin Monoclonal Antibody (YOL1/34)	IgG2a	Thermo Fisher Scientific	MA1-80189
Rabbit polyclonal Anti-AKAP9	IgG	Sigma-Aldrich	HPA026109

### Immunoblots

Whole-cell lysates were generated using the Lysis-M kit solution (cOmplete; Roche) or RIPA buffer (150 mM NaCl, 1% Nonidet P-40 equivalent, 0.5% sodium deoxycholate, 0.1% sodium dodecyl sulphate and 50 mM Tris, pH 7.4) following harvest, and centrifuged at 14 000*g* for 10 min to pellet cell debris. To extract protein from tissues, small slices of tissue were placed into Lysing Matrix D tubes (MP Biomedicals) and snap frozen in liquid nitrogen. After snap freezing, the tubes were either stored at −80°C or used directly for protein analysis. RIPA buffer or protein extraction buffer [50 mM Tris (pH 7.4), 500 mM NaCl, 0.4% sodium dodecyl sulphate (SDS), 5 mM EDTA (pH 7.4), 1× Protease inhibitor (cOmpleteTM EDTA-free Protease Inhibitor cocktail, Cat no. 04693159001, Roche), 2% Triton, 1 mM Dithiothreitol, in deionized water] was added to tissues, which were then homogenized using the FastPrep-24 Instrument (MP Biomedicals). Samples were then centrifuged at 14 000*g* for 10 min to remove cell debris. Protein concentration was quantified using a bicinchoninic acid protein quantification kit (Pierce). Protein samples were resolved by sodium dodecyl sulphate-polyacrylamide gel electrophoresis analysis and transferred to polyvinylidene fluoride membrane (Millipore) by wet transfer for 48 h at 20 V at 4°C. Membranes were blocked in Tris-buffered saline (TBS) containing 0.1% Tween 20 (TBST) supplemented with 5% milk powder (Anlene) for 1 h at room temperature. Western blot analysis was performed using primary antibodies diluted in 5% milk powder, diluted in TBST. Nesprin-1 was immunoblotted using mouse monoclonal anti-Nesprin-1, clone MANNES1E or rabbit monoclonal anti-Nesprin-1, EPR14196. Membranes were incubated for 2 h at room temperature or overnight at 4°C. For secondary antibodies, horseradish peroxidase (Invitrogen) conjugated antibodies were used for chemiluminescent imaging. The membranes were incubated for 1 h at room temperature with the secondary antibodies. For immunoblots visualized by chemiluminescence, membranes were incubated in enhanced chemiluminescence (ECL) substrate (Pierce) for 1 min before being exposed to a chemiluminescence sensitive film (Thermo Scientific) and subsequently processed.

### Immunofluorescence microscopy and image analysis

Cells were grown in eight-well slides (Ibidi), 96-well glass-bottom plates (Ibidi) or coverslips and fixed either in ice-cold methanol for 15 min at −20°C or in 4% paraformaldehyde. They were then rinsed in phosphate-buffered saline (PBS) twice and permeabilized and blocked with 0.1% Triton X-100, 3% bovine serum albumin (BSA) in PBS for 15 min at room temperature. The fixed and permeabilized cells were then rinsed in PBS three times. Samples were then incubated with primary antibodies (Table 1) for 1–2 h at room temperature or overnight at 4°C. Samples were then washed with PBS three times and subsequently incubated with secondary antibodies (Life Technologies) and DAPI (4′,6-diamidino-2-phenylindole, Life Technologies) for 30 min to 1 h at room temperature. After three washes in PBS, cells were mounted in an antifade solution (1% DABCO, 90% Glycerol, 10% PBS) or Prolong Diamond mountant (Invitrogen) and inspected using a Zeiss 510 Meta Confocal microscope or Axiovert 200 inverted epifluorescence microscope (Zeiss) or Olympus FV3000RS confocal microscope. Images were recorded and analysed using Zeiss ZEN, Olympus CellSens, Metamorph or Fiji software.

### Echocardiography

Cardiac function of mice was measured by echocardiography using a Vevo 3100 imaging system 3 weeks after the injection of tamoxifen. The abdomens of the mice were shaved the day before the ultrasound scan to expose the skin. During the procedure, 1.5–2% isoflurane mixed with oxygen was used to anaesthetize the mice. Cardiac package was used to obtain B-mode and M-mode scans with heart rate maintained around 400–500 bpm. Post-processing measurement was done using VevoLAB software (FUJIFILM VisualSonics). Left ventricular function was assessed via tracings of the left ventricle from at least four cardiac cycles, and EF and FS were obtained from short-axis mode.

### Image analysis

To measure nuclear roundness, DAPI images were first thresholded using the Otsu method (Fiji) followed by automatic watershedding algorithm. For isolated cardiomyocytes, nuclear boundaries were delineated by hand. PCNT distribution around nuclei was performed as indicated in Supplementary Material, Figure S3. To evaluate the correlation between nuclear roundness and Pcnt signal distribution, we plotted the overall variation of Pcnt around the perimeter of each nucleus.

### Graphing and statistical analysis

All statistical analysis was performed using Graphpad Prism or Microscoft Excel software. Normal probability density function was determined using NORM.DIST function in Excel. Survival curves were drawn using the Kaplan–Meier method in Prism.

## Supplementary Material

Supp_Figure_1_ddac179

Supp_Figure_2_ddac179

Supp_Figure_3_ddac179

Supp_Figure_4_ddac179

HMG-2021-CE-00873R2-Leong-Nesprin1-SupplementaryLegends_ddac179

## Notes

Present address: Nuevocor Pte Ltd, 3 Biopolis Drive, #06-11 Synapse, Singapore 138623, Singapore.
