# Supplementary figures and images for: Nesprin-1 LINC complexes recruit microtubule cytoskeleton proteins and drive pathology in Lmna-mutant striated muscle
Source: Hum Mol Genet. 2022 Aug 4;32(2):177–91. doi: 10.1093/hmg/ddac179 (PMC9840208; doi:10.1093/hmg/ddac179)

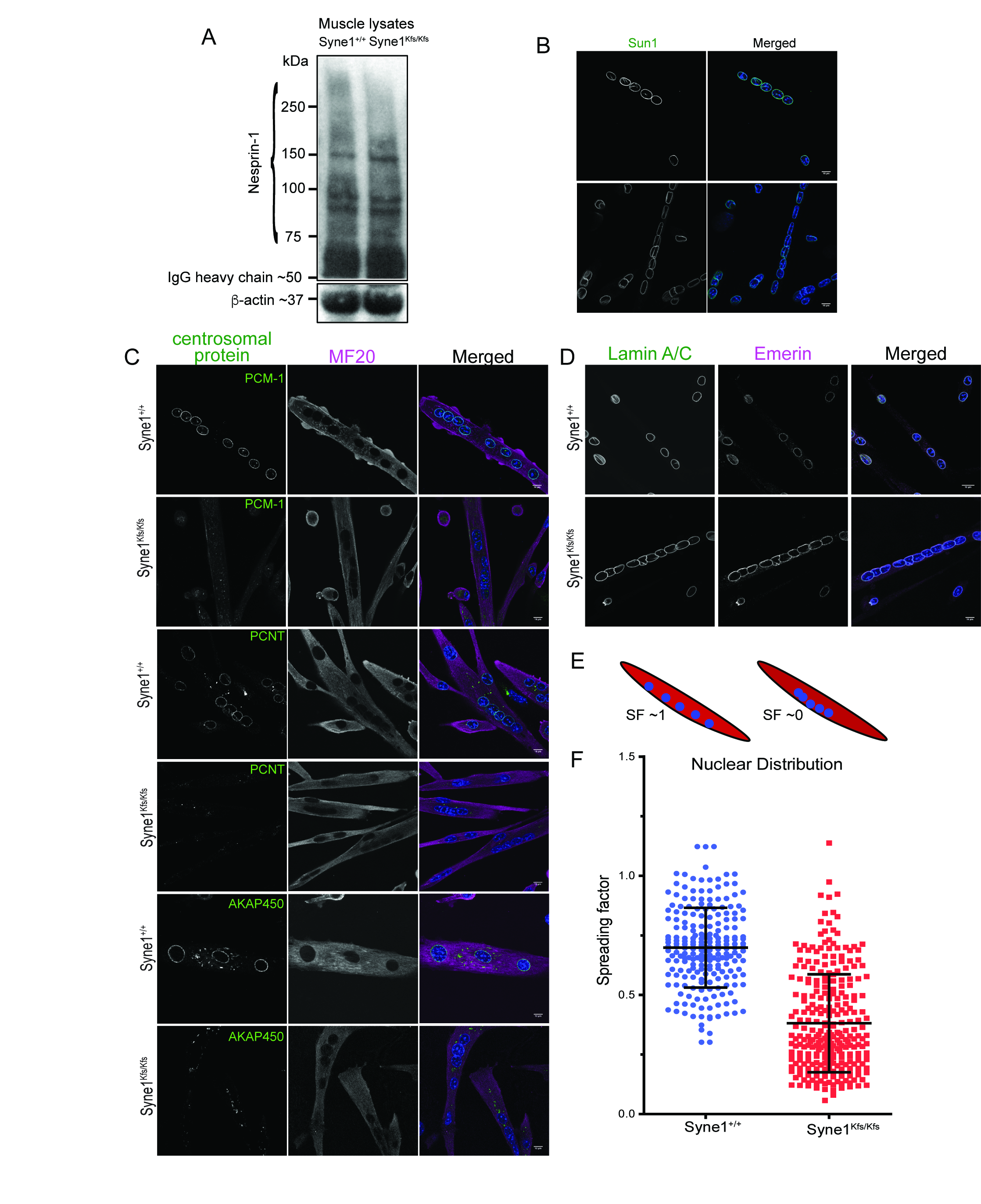

Supplement: Supp_Figure_1_ddac179 [file supp_figure_1_ddac179.zip › Supp_Figure_1_ddac179.tif]

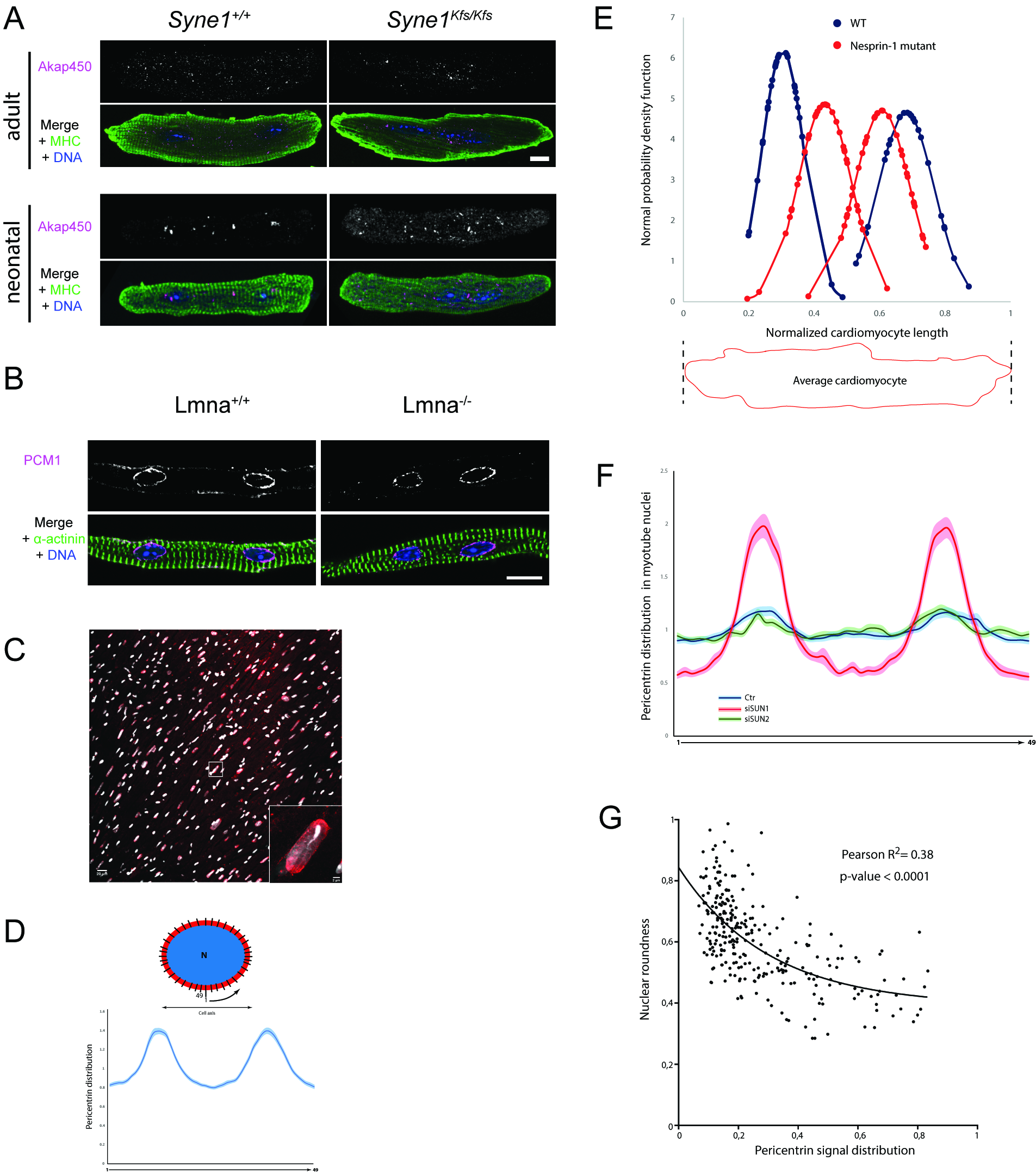

Supplement: Supp_Figure_2_ddac179 [file supp_figure_2_ddac179.zip › Supp_Figure_2_ddac179.tif]

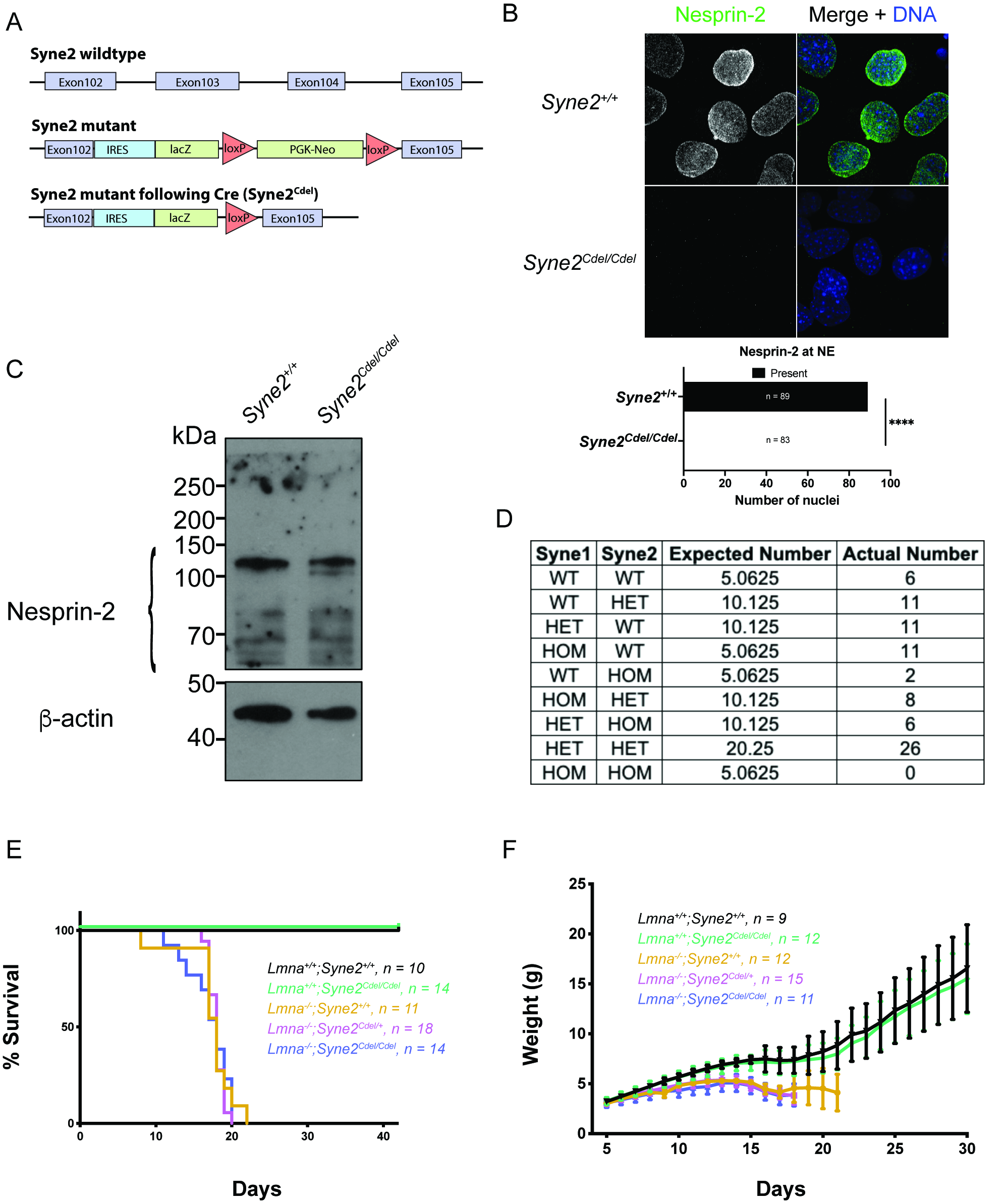

Supplement: Supp_Figure_3_ddac179 [file supp_figure_3_ddac179.zip › Supp_Figure_3_ddac179.tif]

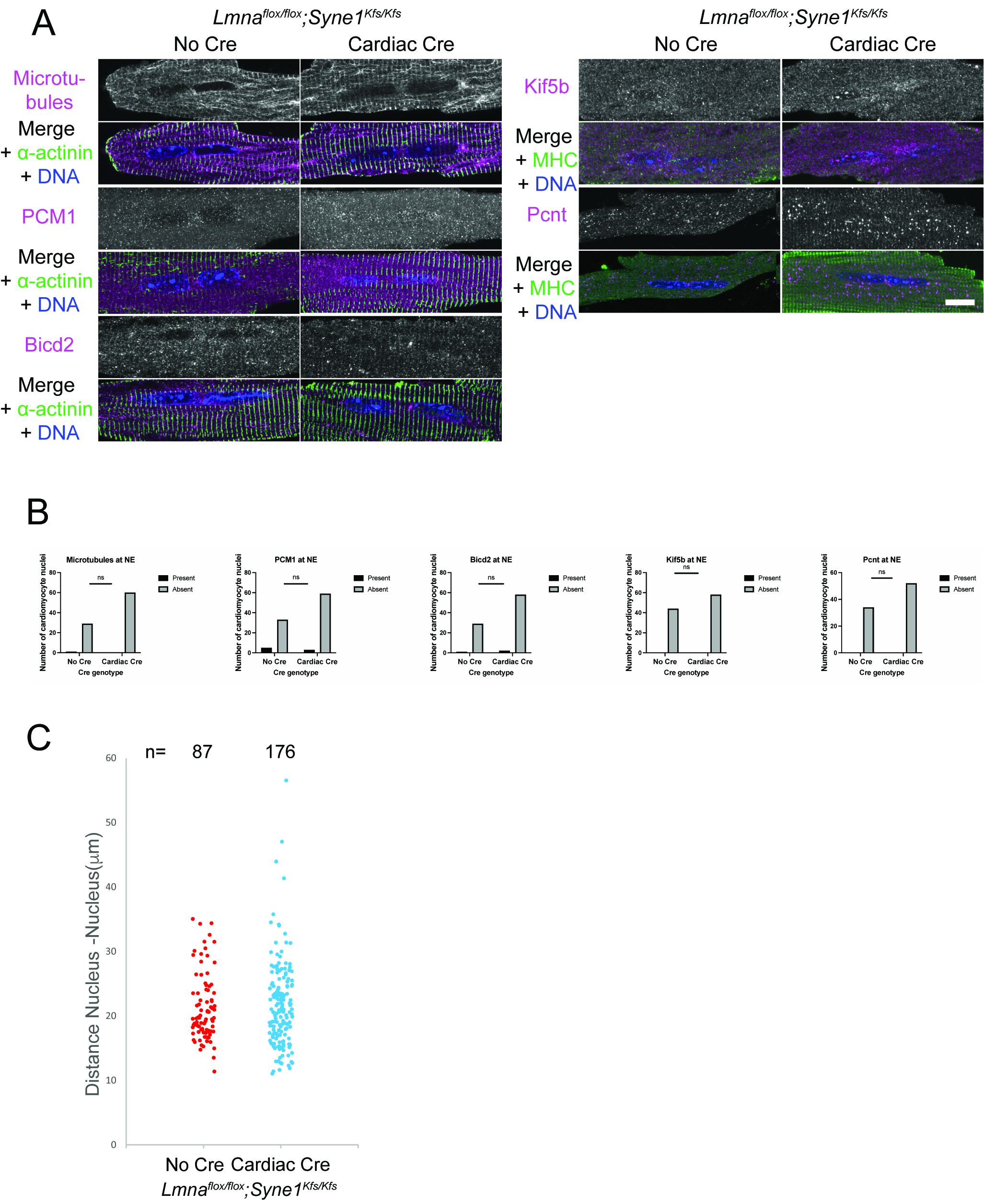

Supplement: Supp_Figure_4_ddac179 [file supp_figure_4_ddac179.zip › Supp_Figure_4_ddac179.tif]
