# Supplementary material for: Nesprin-1 LINC complexes recruit microtubule cytoskeleton proteins and drive pathology in Lmna-mutant striated muscle
Source: Hum Mol Genet. 2022 Aug 4;32(2):177–91. doi: 10.1093/hmg/ddac179 (PMC9840208; doi:10.1093/hmg/ddac179)
Supplement: HMG-2021-CE-00873R2-Leong-Nesprin1-SupplementaryLegends_ddac179 [file hmg-2021-ce-00873r2-leong-nesprin1-supplementarylegends_ddac179.docx]

**Nesprin-1 LINC complexes recruit microtubule cytoskeleton proteins and drive pathology in *Lmna* mutant striated muscle**

**Supplementary Legends**

**Supplementary Figure 1. Nesprin-1 mutation disrupts MTOC protein localization in myotubes**

Characterization of muscle cells from *Syne1^+/+^* and *Syne1^Kfs/Kfs^* mutant mice (A) Muscle tissue dissected from *Syne1^+/+^* and *Syne1^Kfs/Kfs^* mutant mice. Tissue lysates were analyzed by Western blot using antibodies against Nesprin-1 and β-actin. (B) Immunofluorescence microscopy of primary myoblasts isolated from *Syne1^+/+^* and *Syne1^Kfs/Kfs^* mutant mice and then differentiated to form myotubes. Myotubes immunostained for Sun1 (green). (C) *Syne1^+/+^* and *Syne1^Kfs/Kfs^* mutant myotubes immunostained for PCM-1, Pcnt, or AKAP450 (green) and myosin heavy chain (MF20, magenta). (D) *Syne1^+/+^* and *Syne1^Kfs/Kfs^* mutant myotubes immunostained for lamin A/C (green) and emerin (magenta). In (B – D), DNA (blue) was revealed by staining with Hoechst dye. (E) Schematic of spreading factor assay for nuclear positioning in myotubes. The spreading factor represents the quotient of the average internuclear distance and the ideal internuclear distance if the nuclei were uniformly distributed along the myotube length. (F) Spreading factor analysis of nuclei in *Syne1^+/+^* and *Syne1^Kfs/Kfs^* myotubes. Results are depicted as mean with interquartile range, p < 0.0016, unpaired T-test. Clearly, nuclei are clustered in the *Syne1^Kfs/Kfs^* myotubes.

**Supplementary Figure 2. Microtubule cytoskeleton proteins in cardiomyocytes**

Immunofluorescence microscopy of cardiomyocytes isolated from 6 to 8-week-old adult and 10 - 12 day old neonatal wildtype and *Syne1^Kfs/Kfs^* mice (A) and immunostained for AKAP450 (magenta) and myosin heavy chain (MHC, green). Immunofluorescence microscopy of cardiomyocytes isolated from 10-day-old *Lmna^+/+^* and *Lmna^-/-^* mice (B) and immunostained for PCM1 (magenta) and α-actinin (green). Bar, 10 μm. (C) Immunofluorescence microscopy of a wild-type mouse heart section labelled with an antibody against Pcnt. This reveals a polar distribution of Pcnt on cardiomyocyte NEs. DNA (white) is revealed by staining with Hoechst dye Bar, 20 μm. An example of this polarization is visible in the high resolution inset: this represents a projection of 19 Z-plane images with a 0.1 μm step. Bar is 2μm. In both (A) and (B) Representative images of binucleated cardiomyocytes are shown with DNA (blue) revealed by staining with Hoechst dye. (D) Top: To quantify Pcnt distribution around the nuclear periphery, a line 3 μm wide was drawn around multiple nuclei. Starting at a point on the flattened nuclear surface that lies parallel to the long axi of the cell, the line was divided in to 49 segments. In each segment the Pcnt signal was measured and normalized for each nucleus. Bottom: Graph representing Pcnt distribution in 72 nuclei in 3 different hearts. The darker line represents the averaged Pcnt intensity, while the lighter surrounding area displays the SEM. (E) Normal probability density function of nuclear position was plotted against the normalized length of an average cardiomyocyte, revealing longer spacing between nuclei in wildtype (blue line) compared to *Syne1^Kfs/Kfs^* (red line) binucleated cardiomyocytes. (F) Graph representing nuclear envelope Pcnt distribution of at least 55 myotube nuclei from 3 different experiments following control, Sun1 or Sun2 siRNA treatment. Darker line is the mean value, light surrounding area is the SEM. It is clear that the polar distribution of Pcnt (Supplemental Figure 2) is greatly enhanced in cells depleted of Sun1 but not Sun2. (G) Correlation plot between nuclear roundness and Pcnt signal distribution around the nucleus indicates a relationship between Pcnt localisation and nuclear shape. P-value was determined by t-test.

**Supplementary Figure 3. Nesprin-2 C-terminal deletion is synthetic lethal with Nesprin-1 KASH deletion but does not suppress lamin A/C mutation.**

(A) Schematic showing gene targeting of *Syne2* to generate a *Syne2^Cdel^* mutant allele. The deletion encompasses spectrin repeats 51-53 in Nesprin-2 giant. (B) Fibroblasts were isolated from skeletal muscle of *Syne2^+/+^* and *Syne2^Cdel/Cdel^* mice, fixed and immunostained for Nesprin-2 (green) and counterstained for DNA (blue) with Hoechst dye. Clearly, Nesprin-2 is absent from the NE in mutant cells, and this is quantified in the graph. Statistical significance was determined by Fisher’s exact test. ****, P-value < 0.0001. (C) Fibroblasts from *Syne2^+/+^* and *Syne2^Cdel/Cdel^* mice were lysed and analyzed by Western blot using Nesprin-2 and α-actin antibodies. Multiple Nesprin-2 isoforms are present in both cell poulations. However, these are not NE associated in the fibronlasts derived from the *Syne2^Cdel/Cdel^* mice (A). (D) Table summarizing expected and observed offspring from dihybrid cross of *Syne1^+/Kfs^;Syne2^+/Cdel^* mice, χ^2^ = 18.0, p = 0.022. A total of 81 pups were genotyped after weaning. No double mutant mice were observed, consistent with previously reported perinatal lethality. (E, F) *Lmna^+/-^;Syne2^+/Cdel^* mice were intercrossed to obtain *Lmna^+/+^;Syne2^+/+^* , *Lmna^+/+^;Syne2^Cdel/Cdel^* , *Lmna^-/-^;Syne2^+/+^ , Lmna^-/-^;Syne2^Cdel/Cdel^* mice and their survival (E) and weight (F) were monitored for up to 40 days. The Nesprin-2 mutation failed to rescue the *Lmna* null mutation either in terms of life span or weight loss.

**Supplementary Figure 4. Disruption of Nesprin-1 KASH domain results in loss of microtubule-associated proteins and nuclear positioning regardless of *Lmna* genotype.** Immunofluorescence microscopy of cardiomyocytes isolated from 6-8 week old mice. Cardiomyocytes were isolated from *Lmna^flox/flox^;Syne1^Kfs/Kfs^* mice lacking (No Cre) or harbouring *Tg(Myh6-cre/Esr1*)* (Cardiac Cre) following treatment with tamoxifen*.* (A) Cardiomyocytes were immunostained for microtubules, kinesin heavy chain (Kif5b), PCM1, Pcnt (Pcnt), or Bicd2 (all in magenta) and α-actinin (green) or myosin heavy chain (MHC, green). Representative images of binucleated cardiomyocytes are shown. (B) Quantification of number of cardiomyocyte nuclei lacking (No Cre) or harbouring *Tg(Myh6-cre/Esr1*)* (Cardiac Cre) with protein of interest present at (black) or absent from (grey) the nuclear envelope (NE). Fisher’s exact test was used to determine statistical significance – ns, not significant.
